# Variance Resonance in Weakly Coupled Harmonic Oscillators Driven by Thermal Gradients

**DOI:** 10.3390/e26121087

**Published:** 2024-12-12

**Authors:** Tarcisio Boffi, Paolo De Gregorio

**Affiliations:** 1 Independent Researcher, 00179 Rome, Italy; tarcisioboffi@gmail.com; 2 Dipartimento di Scienze Matematiche, Politecnico di Torino, Corso Duca degli Abruzzi 24, 10129 Torino, Italy

**Keywords:** nonequilibrium steady states, local equilibrium, dissipative systems, precision measurements, resonant systems

## Abstract

We study two harmonic oscillators with high quality factors, driven by equilibrium and off equilibrium thermal noise, the latter mimicked by establishing a temperature gradient. The two oscillators are coupled via a third reciprocal harmonic interaction. We deepen the case of a weak coupling between the two oscillators, and show the emergence of a “spike” in the displacement variance of the colder oscillator, when the respective elastic constants approach each other. Away from the peak, the displacement variance of each oscillator only reflects the value of the local temperature. We name this phenomenon the variance resonance, or alternatively covariance resonance, in the sense that it comes about as one element of the covariance matrix describing both oscillators. In fact, all of the elements of the covariance matrix show some distinctive behavior. The oscillator at the lower temperature, therefore, oscillates as if driven by a higher temperature, resonating with the other one. By converse, the variance of the hotter oscillator develops a deep dent, or depression, around the same region. We could not reproduce this behavior if either the coupling constant is not small compared to those of the two oscillators, or if the quality factors are not large enough. In fact, in such instances the system tends to resemble one which is in equilibrium at the average temperature, regardless of the relative strengths of the elastic constants of the two oscillators. Our results could have various applications including for example precision measurement systems, when not all parts of the apparatuses are at the same temperature.

## 1. Introduction

In precision measurements, the detector (or detectors) are often modeled in a first approximation as harmonic oscillators with high quality factors [1,2,3,4,5,6]. Each oscillator represents one specific (known) mode of vibration of the device. The magnitude of the quality factors implies that the loss of energy due to thermo-mechanical dissipation, around each mode of oscillation, takes place over several cycles (from thousands to even hundreds of millions). Examples can be mirrors suspended by thin wires [7], or cantilevers [1,2,8]. Such devices are particularly sensitive to excitations which occur, in frequency, in the proximity of the resonance frequency of any given measurable mode of vibration of the object. It is thus important to know in advance, and be able to control, the “baseline” of the vibrating behavior, i.e., when no signal is being observed. Intrinsic exciting forces can be naturally occurring seismic movements, thermal excitations and the like.

In recent years, there has been a considerable interest in studying the effects of temperature gradients on precision measurement devices [4,5,9]. It is not always possible to guarantee that the whole system is, in fact, in thermal equilibrium, and there can be parts hotter or colder than others. Our model is so simple that it can even be used a posteriori to illustrate such point, in that it shows in principle that one part of the system may behave as if standing at a different temperature than the one measured locally. Of course, in a realistic situation one would have to enrich the mathematical model according to the specific experimental setup, but we expect such enrichment to extrapolate the same basic phenomenon, at a more quantitative level.

**Figure 1:**
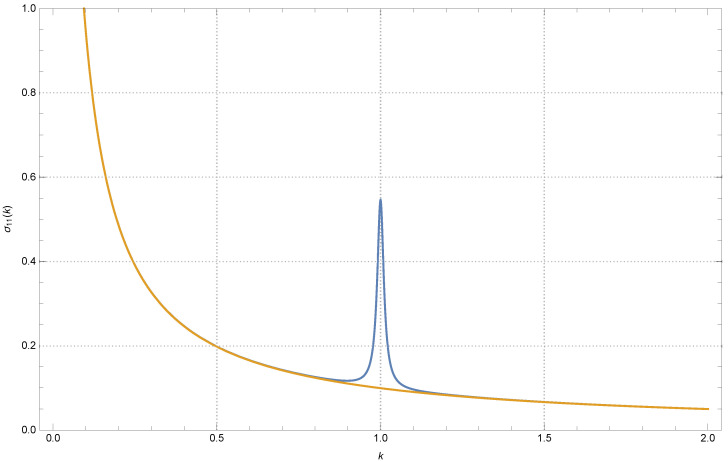
The variance $\sigma_{11}$ of the first oscillator (in arbitrary units), as a function of $k=k_{1}/k_{2}$ (the ratio of the oscillators’ elastic constants). In blue, presenting a peak around k=1, the case $T_{1}<T_{2}$. The orange monotonic curve represents $T_{2}$ lowered at the value $T_{2}=T_{1}$.

To put things into perspective, we can consider a system of several cantilevers used at once, in parallel. The power spectra of such cantilevers have been thoroughly studied, both experimentally and theoretically (see for example [1]). Usually, due to unavoidable imperfections in their construction, the cantilevers are not totally identical to one another, e.g., their elastic moduli may differ by a few small fractions (in relative terms). Let us consider the case of a system of two cantilevers. In first approximation, such a system has been studied as a system of weakly coupled harmonic oscillators. Much is known about their response to harmonic forcing [1]. Here, we concentrate instead on the case in which the forcing is only thermal, comparing the equilibrium case to the nonequilibrium case (i.e., the two oscillators are driven by stochastic thermostats at different temperatures). Any of the two model’s oscillators has its own elastic force that tends to keep it in place, which represents a single intrinsic mode of vibration. Additionally, the two oscillators also interact elastically, albeit weakly. It is just a model of the “modal” coupling that occurs, e.g., via some shared supporting element in the apparatus. We are going to show that, out of equilibrium and in particular circumstances, there is a sort of interference between the two oscillators, which becomes noticeable only when the two oscillators have nearly the same elastic constants. We call this phenomenon “variance resonance” or “covariance resonance”. More precisely, if we call $\sigma_{11}=\langle {\left(x_{1}-\langle x_{1}\rangle \right)}^{2}\rangle$ the variance of the first oscillator at $T_{1}<T_{2}$, and plot it as a function of $k=k_{1}/k_{2}$, i.e., the ratio of the elastic constants, we discover a peak around k=1. This trend is illustrated in Figure 1, where it is compared to the equilibrium curve, $T_{1}=T_{2}$.

Systems similar to ours have been well studied in the past, e.g., chains of oscillators in the presence of thermal gradients [10]. Pairs of oscillators have also been studied in depth, and often the interest has been in the overdamped case, i.e., for very low quality factors [11,12,13]. We are interested in the case of very high quality factors, i.e., the (strongly) underdamped case. We are therefore less interested in the mechanisms of energy dissipation. It is also worth noticing that the phenomenon we observe is unrelated to that of stochastic resonance [14,15]. In fact, the potential well in our case is not bimodal or multimodal, while stochastic resonance first emerges in bistable systems. Additionally, the peak of the covariance that we observe emerges as a function of a structural variable, the ratio of elastic constants, and not of the frequency. For this very reason, however, we found the phenomenon worthy of investigation. In essence, such “resonance” can be observed by concentrating on a single oscillator as a subpart of a collective system. It is an internal redistribution of energy driven by the thermal gradient, and mediated by the weak elastic coupling.

**Figure 2:**
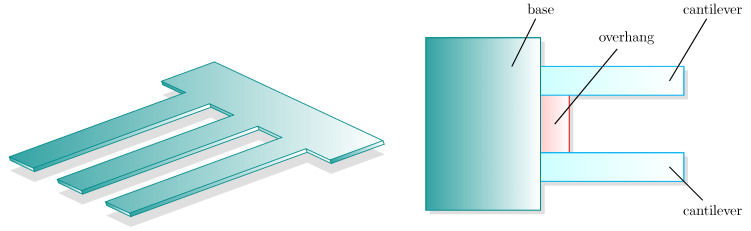
Systems of cantilevers. On the left, a simplified ideal representation. Typically, the flexural modes can be detected. As in the right picture, a rigid overhang may be present as an additional mechanical element, causing unwanted tiny communications between the modes.

A schematic depiction of a system of two or three cantilevers is presented in Figure 2.

## 2. Definition of the Model

In a classically driven damped harmonic oscillator,

(1) $$\begin{array}{c} \ddot{x}+\beta \dot{x}+\omega_{0}^{2}x=A\cos\left(\omega t\right) \end{array}$$

whose stationary solution reads

(2) $$\begin{array}{c} x(t)=|c(\omega )|\cos[\omega t-\varphi ] \end{array}$$

where φ is a phase factor and |c(ω)| the oscillation amplitude, |c(ω)| shows a resonance peak when $\omega \to \omega_{0}\sqrt{1-\zeta^{2}/2}$, where $\zeta =\beta /\omega_{0}$ (provided $\sqrt{2}\;\zeta <1$). For very small ζ, then the resonance occurs very approximately at $\omega \to \omega_{0}$, the undamped case.

In the case of a stochastic driving, Kramers’ equation reads,

(3) $$\begin{array}{c} \ddot{x}+\beta \dot{x}+\omega_{0}^{2}x=\sqrt{D}\xi \left(t\right) \end{array}$$

where ξ(t) is a Gaussian white noise, i.e., $\langle \xi \left(t\right)\xi \left(t^{\prime }\right)\rangle =\delta \left(t-t^{\prime }\right)$, and $\sqrt{D}=\sqrt{2\beta k_{B}T/m}$ ($k_{B}T$ the Boltzmann constant, *T* the absolute temperature and *m* the mass of the oscillating body). Since Equation (3) is linear and ξ(t) white and Gaussian, the solution x(t) is a generalized Gaussian process (with finite two-time correlators), unlike (2). The driving injects all frequencies with equal weights, and not just a single component at frequency ω. The resonant character of the oscillator only emerges by analyzing the averaged frequency spectrum of the underlying dynamics, which is a Lorentzian peaked at the same value $\omega ≃\omega_{0}$. Nevertheless, the analogy weakens by looking at the mere amplitudes of oscillations, when considering the relevant meaningful quantities in the two cases,

(4) $$\begin{array}{c} {\mathrm{Deterministic}\mathrm{case}:|c\left(\omega \right)|}^{2}\;↔\;\mathrm{Thermal}\mathrm{case}:\langle x,x\rangle :=\langle {\left(x-\langle x\rangle \right)}^{2}\rangle \end{array}$$

For simplicity, we have assumed 〈x〉=0, i.e., $\langle x,x\rangle \equiv \langle x^{2}\rangle$ (equal times). Both quantities are a measure of the mean squared displacement from the equilibrium position, but in the thermal case we have

(5) $$\begin{array}{c} \langle x^{2}\rangle =\frac{k_{B}T}{k} \end{array}$$

a constant value, which can be rewritten as a function of $\omega_{0}=\sqrt{k/m}$ as:

(6) $$\begin{array}{c} \langle x^{2}\left(\omega_{0}\right)\rangle =\frac{k_{B}T}{m\omega_{0}^{2}} \end{array}$$

What happens if we act on *k* or on $\omega_{0}$ (which is the same), instead of the driving frequency ω? In the deterministic case, ${\left|c\left(\omega \right)\right|}^{2}$ would display its resonant character when $\omega_{0}$ crosses the region in the proximity of ω. Of course, conversely, by Equation (6), the thermal case displays no resonance, and the variance of *x* is just a monotonic function of *k* or $\omega_{0}$. Then, the question arises. Suppose that for a thermally driven system the variance of some stochastic observable *x* displays a peak around some hypothetical value $\omega_{0}≃\omega_{r}$. It is in this sense, by loose analogy, that we shall talk about variance or covariance resonance (when it involves more than one oscillating element).

**Figure 3:**
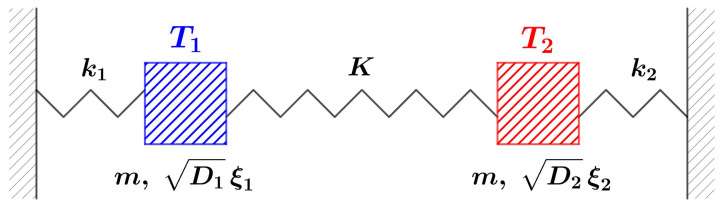
Illustrative representation of our model, Equation (8).

Our model, schematically illustrated in Figure 3, involves two damped harmonic oscillators, both of mass *m*, with elastic forces with constants $k_{1}$ and $k_{2}$ pinning them to their rest positions $x_{1}=0$ and $x_{2}=0$. Additionally, a third elastic force of constant *K* is exerted between the two, with rest position $x_{1}=x_{2}$. The damping is $\gamma {\dot{x}}_{i}$ for both oscillators. The dynamics of our model read

(7) $$\begin{array}{c} \left\{\begin{array}{c} m{\ddot{x}}_{1}=-\partial_{x}V\left(x,y\right)-\gamma {\dot{x}}_{1}+\sqrt{D_{1}}\xi_{1}\\ m{\ddot{x}}_{2}=-\partial_{y}V\left(x,y\right)-\gamma {\dot{x}}_{2}+\sqrt{D_{2}}\xi_{2} \end{array}\right. \end{array}$$

with $V\left(x,y\right)=\frac{k_{1}}{2}x_{1}^{2}+\frac{k_{2}}{2}x_{2}^{2}+\frac{K}{2}{\left(x_{1}-x_{2}\right)}^{2}$. The equations of motion read

(8) $$\begin{array}{c} \left\{\begin{array}{c} m{\ddot{x}}_{1}=-k_{1}x_{1}-\gamma {\dot{x}}_{1}-K\left(x_{1}-x_{2}\right)+\sqrt{D_{1}}\xi_{1}\\ m{\ddot{x}}_{2}=-k_{2}x_{2}-\gamma {\dot{x}}_{2}-K\left(x_{2}-x_{1}\right)+\sqrt{D_{2}}\xi_{2} \end{array}\right. \end{array}$$

where $\sqrt{D_{i}}=\sqrt{2\gamma k_{b}T_{i}}$, while $\xi_{1}$ and $\xi_{2}$ are standard Gaussian white noises.

Similar systems have been studied before, solely or with more emphasis on the underlying overdamped limit [11,12,13], and/or with a slightly different potential [16,17,18]. Most often, the considered differential equations of motion turn out to be coupled, but of the first order. On the contrary, we suppose here that $mk_{i}\gg \gamma^{2}$, the underdamped case, and the coupled equations of motion must remain of second order at the source. In general, we suppose $k_{1}$ and $k_{2}$ to be at least of comparable orders of magnitude. The strength of *K* relative to the $k_{i}$’s determines the extent of the coupling. When comparable, the system is essentially equivalent to a thermostatted two-particle system, interacting elastically almost identically—both between themselves and with two fixed boundaries at the opposite ends. When $K\gg \max\left(k_{i},k_{2}\right)$, the boundary interaction is negligible. When $K\ll \min\left(k_{i},k_{2}\right)$, the two particles oscillate almost independently, but they are also weakly coupled with each other, over a characteristic time much larger than those of the two individual oscillations. It is not unreasonable to suppose that such scenario, albeit very simplistically reduced here, might emerge when two vibrating small-sized objects are not entirely isolated, but share some interaction with a larger object. In fact, the condition $K\ll \max\left(k_{i},k_{2}\right)$ might also reflect the effect of a reduced elastic constant *K*, which carries also information about the large mass of the third body, compared to the smaller vibrating masses. The analogy should not be taken too literally, since our model merely aims to mimic some interaction between specific observable modes of vibration, possibly among enumerably infinite ones. We point out that a system similar to that of Equations (8), but with deterministic forcing in place of the stochastic ones, has been proposed to study the so-called Fano classical resonances between several cantilevers in parallel [1]. The rationale for such simplification is to study separately the cantilevers pair by pair, selecting each time those which are closest in terms of intrinsic resonance frequencies, since the effect of those which are further away in resonances is less prominent. Additionally, the fact that all of the cantilevers have very high quality factors renders the collective effects reasonably well approximated by the sum of independent pair processes. In this work, we shall adopt the same approach, because it is the most immediately informative and more easily analytically solvable. We are well aware that a finer treatment would require a system of equations larger than (8), whose space of parameters to explore however would grow substantially and it is beyond the scope of the present treatment, which aims to show a new phenomenon in the simplest approximation.

## 3. Towards Dimensionless Variables

In what follows, we shall rewrite the equations introducing some dimensionless quantities. This will make the treatment both more transparent and exportable to differing situations. Our aim is to render the equations of motion dimensionless themselves. It is best to first introduce the transformation of our interest for a single harmonic oscillator, and only next to rewrite the model’s equations of motion.

Kramers’ equation for a single harmonic oscillator reads

(9) $$\begin{array}{c} mx_{tt}=-kx-\gamma x_{t}+F \end{array}$$

where *F* is just the as-yet-unspecified driving. The subscript for the differential operators is motivated by the fact that we are going to also transform the independent variable *t* into a dimensionless one, *s*. We want to preserve the usual representation with dots for the latter variable now.

Call *L* a characteristic length of the system, e.g., the length of a cantilever. Call τ a characteristic time of the system, e.g., the inverse of the resonance frequency of the fundamental mode of vibration of the cantilever. We can thus rewrite Equation (9) using the following dimensionless quantities *u* and *s*, where

(10) $$\begin{array}{cc} x & =Lu\Rightarrow u\mathrm{is}\mathrm{adimensional} \end{array}$$

(11) $$\begin{array}{cc} t & =s\tau \Rightarrow s\mathrm{is}\mathrm{adimensional} \end{array}$$

It follows that

(12) $$\begin{array}{cc} \frac{\partial }{\partial t} & =\frac{1}{\tau }\;\frac{\partial }{\partial s} \end{array}$$

(13) $$\begin{array}{cc} \frac{\partial^{2}}{\partial t^{2}} & =\frac{1}{\tau^{2}}\frac{\partial^{2}}{\partial s^{2}} \end{array}$$

and using now the notation $\frac{du(s)}{ds}=\dot{u}$, Equation (9) can be rewritten as

(14) $$\begin{array}{c} \ddot{u}+\frac{\gamma \;\tau }{m}\dot{u}+\frac{k\;\tau^{2}}{m}u=\frac{F\;\tau^{2}}{m\;L} \end{array}$$

If we now choose $\tau =\frac{1}{\omega_{0}}=\sqrt{\frac{m}{k}}$, we obtain

(15) $$\begin{array}{c} \ddot{u}+\frac{1}{Q}\dot{u}+u=\frac{F}{kL} \end{array}$$

where $Q=\frac{\sqrt{km}}{\gamma }$ is the quality factor of the oscillator.

We repeat the same reasoning to also transform the system given by (8) into an equivalent dimensionless one. It can be rewritten as follows:

(16) $$\begin{array}{c} \left\{\begin{array}{c} {\ddot{u}}_{1}+\frac{k_{1}\;\tau^{2}}{m}u_{1}+\frac{\gamma \tau }{m}{\dot{u}}_{1}+\frac{K\;\tau^{2}}{m}\left(u_{1}-u_{2}\right)=\frac{\sqrt{D_{1}}\;\tau^{2}}{m\;L}\xi_{1}\\ {\ddot{u}}_{2}+\frac{k_{2}\;\tau^{2}}{m}u_{2}+\frac{\gamma \tau }{m}{\dot{u}}_{2}+\frac{K\;\tau^{2}}{m}\left(u_{2}-u_{1}\right)=\frac{\sqrt{D_{2}}\;\tau^{2}}{m\;L}\xi_{2} \end{array}\right. \end{array}$$

We could set τ to be the inverse of either natural frequencies. In our case, we choose $\tau =\sqrt{\frac{m}{k_{2}}}$. We define the following quantities:

(17) $$\begin{array}{cc} Q_{2} & =\frac{\sqrt{k_{2}m}}{\gamma }\;\left(\mathrm{quality}\mathrm{factor}\mathrm{of}\mathrm{the}\mathrm{second}\mathrm{oscillator}\right) \end{array}$$

(18) $$\begin{array}{cc} k & =\frac{k_{1}}{k_{2}} \end{array}$$

(19) $$\begin{array}{cc} k_{0} & =\frac{K}{k_{2}} \end{array}$$

Furthermore, we define

(20) $$\begin{array}{c} D_{i}=\frac{2}{Q_{2}}\frac{T_{i}}{T_{0}} \end{array}$$

where $T_{0}$ is a characteristic temperature of the system, defined as $T_{0}=\frac{m\;L^{2}}{k_{B}\;\tau^{2}}=\frac{k_{2}\;L^{2}}{k_{B}}$.

System (16) can be rewritten as (if $\eta_{i}=\sqrt{\tau }\;\xi_{i}$ are dimensionless white noises)

(21) $$\begin{array}{c} \left\{\begin{array}{c} {\ddot{u}}_{1}+\left(k+k_{0}\right)u_{1}-k_{0}u_{2}+\frac{1}{Q_{2}}{\dot{u}}_{1}=\sqrt{D_{1}}\;\eta_{1}\\ {\ddot{u}}_{2}+\left(1+k_{0}\right)u_{2}-k_{0}u_{1}+\frac{1}{Q_{2}}{\dot{u}}_{2}=\sqrt{D_{2}}\;\eta_{2} \end{array}\right. \end{array}$$

We can rewrite such a system as an Ornstein–Uhlenbeck one (OU), i.e.,

(22) $$\begin{array}{c} \left\{\begin{array}{c} {\dot{u}}_{1}=v_{1}\\ {\dot{u}}_{2}=v_{2}\\ {\dot{v}}_{1}=-\left(k+k_{0}\right)u_{1}+k_{0}u_{2}-\frac{1}{Q_{2}}v_{1}+\sqrt{D_{1}}\;\eta_{1}\\ {\dot{v}}_{2}=-\left(1+k_{0}\right)u_{2}+k_{0}u_{1}-\frac{1}{Q_{2}}v_{2}+\sqrt{D_{2}}\;\eta_{2} \end{array}\right. \end{array}$$

Notice that, in the latter dimensionless expressions, $Q_{2}$ is not only explicit in the equations, but is also implicit in the definition of $D_{i}$, as outlined in (20).

## 4. The Covariance Matrix

Keeping in mind that *s*, as defined in Equation (11), is just proportional to *t*, and a dimensionless time, we define $y\left(s\right)={\left(u_{1},u_{2},v_{1},v_{2}\right)}^{T}$. We can rewrite the system (22) as

(23) dy(s)=−Ay(s)ds+Bη(s)ds

(24) $$\begin{array}{c} A=\left(\begin{array}{cccc} 0 & 0 & -1 & 0\\ 0 & 0 & 0 & -1\\ \left(k+k_{0}\right) & -k_{0} & \frac{1}{Q_{2}} & 0\\ -k_{0} & \left(1+k_{0}\right) & 0 & \frac{1}{Q_{2}} \end{array}\right)\;B=\left(\begin{array}{cccc} 0 & 0 & 0 & 0\\ 0 & 0 & 0 & 0\\ 0 & 0 & \sqrt{D_{1}} & 0\\ 0 & 0 & 0 & \sqrt{D_{2}} \end{array}\right) \end{array}$$

For an OU process, the stationary solution to the Fokker–Planck equation (FP) is

(25) $$p_{s}\left(y\right)=Ne^{-\frac{1}{2}y^{T}\sigma^{-1}y}$$

where the matrix σ is the stationary covariance matrix

(26) $$\sigma ={\langle y\left(s\right),y^{T}\left(s\right)\rangle }_{S}$$

Since, for an OU process, it can be shown that

(27) $$A\sigma +\sigma A^{T}={\mathrm{BB}}^{T}$$

and since we know the matrices A and B, the relation (27) allows us to determine the stationary covariance matrix. The resulting 4×4 matrix bears 7 degrees of freedom,

(28) $$\left(\begin{array}{cccc} \sigma_{11} & \sigma_{21} & 0 & -\sigma_{23}\\ \sigma_{21} & \sigma_{22} & \sigma_{23} & 0\\ 0 & \sigma_{23} & \sigma_{33} & \sigma_{34}\\ -\sigma_{23} & 0 & \sigma_{34} & \sigma_{44} \end{array}\right)$$

All elements of the matrix σ can be determined, but here we concentrate our attention on the variances $\sigma_{11}$ and $\sigma_{22}$. First, let us consider $\sigma_{11}=\langle u_{1}^{2}\rangle$ explicitly. For brevity, call $Q_{2}=q$ (see (17)). Also, we replace the temperatures $T_{i}$’s with dimensionless ratios, i.e.,

$$T_{i}:=\frac{T_{i}}{T_{0}}$$

The expression for $\sigma_{11}$ is somewhat cumbersome; therefore, we break it into parts, so as to write upon stationarity that

(29) $$\begin{array}{ccc} & \sigma_{11}=\frac{k_{0}^{2}\;T_{2}\;A+T_{1}\;B}{C} & \\ & A=\left(k+1\right)q^{2}+2k_{0}q^{2}+2 & \\ & B={\left(k-1\right)}^{2}q^{2}+k_{0}\left({\left(k-1\right)}^{2}q^{2}+k_{0}\left(-\left(k-3\right)q^{2}+2k_{0}q^{2}+2\right)+2\left(k+3\right)\right)+2\left(k+1\right) & \\ & C=\left(k+\left(k+1\right)k_{0}\right)E;\;E={\left(k-1\right)}^{2}q^{2}+4k_{0}\left(k_{0}q^{2}+1\right)+2\left(k+1\right) & \end{array}$$

When $T_{1}=T_{2}T$, Equation (29) reduces to the formula

(30) $$\begin{array}{c} \sigma_{11}^{\left(eq\right)}\left(T\right)=\frac{\left(k_{0}+1\right)T}{k+\left(k+1\right)k_{0}} \end{array}$$

and Equation (29) (for any combination of temperatures) can be usefully recast as

(31) $$\begin{array}{c} \sigma_{11}=\sigma_{11}^{\left(eq\right)}\left(T_{1}\right)+\sigma_{11}^{\left(neq\right)};\;\;\sigma_{11}^{\left(neq\right)}=\frac{k_{0}^{2}\left(2+q^{2}\left(k+2k_{0}+1\right)\right)\left(T_{2}-T_{1}\right)}{C} \end{array}$$

Setting $T_{1}=T_{2}$ and substituting for σ in Equation (25), we obtain the familiar Maxwell–Boltzmann distribution. Apart from an opportune rescaling of the elastic coefficients, Equation (30) is the same in spirit as Equation (5). The coupling to the second oscillator renormalizes the variance, but no resonance is visible, as one would expect at equilibrium. In particular, $\sigma_{11}$ as a function of $k=k_{2}/k_{1}$, i.e., of the ratio of the elastic constants, is a monotonically decreasing function.

Things turn out to be more interesting when $T_{1}<T_{2}$. For several choices of the parameters, $\sigma_{11}$ behaves very similarly to Equations (5) or (30). Nevertheless, we have found cases in which the interesting phenomenon of covariance resonance presents. We started by setting $q=Q_{2}$ to a reasonably large value, a situation typical of several precision measurements. Recall that $k_{0}=K/k_{2}$ is the ratio between the coupling elastic constant and that of the second oscillator, and it is assumed to take a small value. By decreasing *k*, at some point a local peak starts to develop in the proximity of k=1, which shrinks in width with decreasing *k*. Furthermore, the peak becomes more and more pronounced as the quality factor *q* increases, until a point of saturation. By the point of view of a local observer monitoring oscillator 1, which is at $T_{1}<T_{2}$, suddenly it appears excited, as if at a temperature larger than $T_{1}$. For nearly identical oscillators 1 and 2, the apparent heating is maximum. Differently said, local equilibrium is suddenly broken.

Of course, thermal energy cannot be just created out of nothing. In fact, $\sigma_{22}$ shows a symmetrically opposite behavior. For the same set of parameters, $\sigma_{22}$ is suddenly depressed, as if the second oscillator were at a lower temperature than $T_{2}$. For nearly identical oscillators, the suppression of the oscillations for the hotter oscillator is maximum. Informally, the oscillators “talk” to each other and tend to converge to the average temperature. We are not aware of similar observations in other studies, theoretical or experimental. The key is that the phenomenon depends on a set of parameters.

Our findings will be illustrated in more detail in the next section.

## 5. Results

### 5.1. Variance Resonance

**Figure 4:**
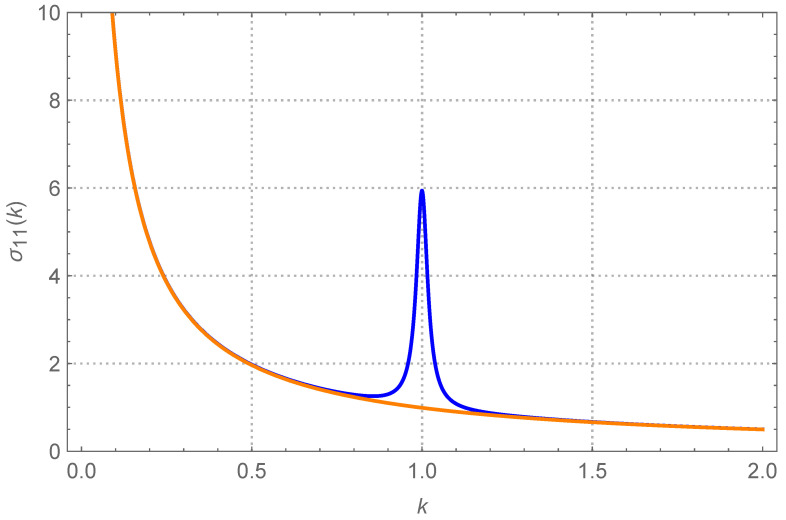
Cold oscillator. The dimensionless stationary $\sigma_{11}=\langle u_{1}^{2}\rangle$ as a function of the ratio $k=k_{1}/k_{2}$. The coupling $k_{0}=K/k_{1}$ is $k_{0}=0.01$. The quality factor $q=Q_{2}$ is q=4000. The blue curve, presenting a peak around k=1, is for $T_{1}=1$ and $T_{2}=11$ (where $T_{i}=T_{i}/T_{0}$). Notice that at k=1, $\sigma_{11}=\left(T_{1}+T_{2}\right)/2$, the average temperature. Further increasing *q* has basically no effect. The orange monotonically decreasing curve is for $T_{1}=T_{2}=1$, i.e., the variance of the first oscillator if the whole system where at its local temperature $T_{1}=1$. Away from the peak the two curves are indistinguishable.

In Figure 4, we report the appearance of the peak of $\sigma_{11}=\langle u_{1}^{2}\rangle$ when q=4000, $T_{1}=1$ and $T_{2}=11$. The coupling is small, k=0.01. In order to understand the significance of such behavior, we compare such curve with the case $T_{1}=T_{2}=1$, all other parameters being equal (the monotonically decreasing curve). Away from k=1, i.e., when the two oscillators have sufficiently different elastic constants, the variance of the first oscillator is therefore indistinguishable from what it would be if the entire system were thermalized at T=1. In other words, the fact that the second oscillator is at a higher temperature has no bearing on the first.

Near k=1, suddenly the peak develops and the variance of the first oscillator approaches a larger value. It is only in this region that the weak coupling bears an effect. Incidentally, exactly at k=1, the (dimensionless) variance of the first oscillator almost equates $\frac{T_{1}+T_{2}}{2}=6$, the dimensionless average temperature. We shall come back to this peculiarity very briefly, but let us anticipate that this is a consequence of the quality factor being very high. Therefore, now the first oscillator oscillates in magnitude as if the whole system were thermalized at the same (eventually average) temperature.

**Figure 5:**
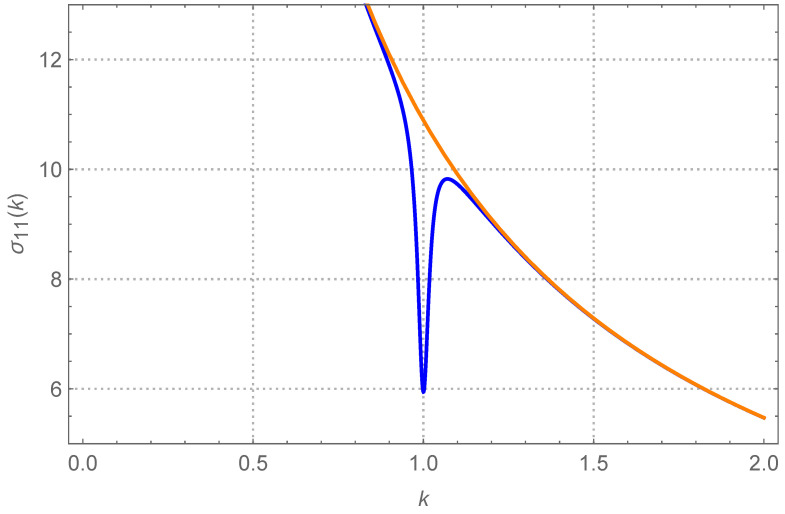
Hot oscillator. The dimensionless stationary $\sigma_{11}$ as a function of the parameter *k*. The coupling is $k_{0}=0.01$. The quality factor is q=4000. The blue curve, presenting a dip (a hollow) around k=1, is for $T_{1}=11$ and $T_{2}=1$. Notice again that at k=1, $\sigma_{11}=\left(T_{1}+T_{2}\right)/2$. The monotonically decreasing curve (in orange) is for $T_{1}=T_{2}=11$, i.e., it is the variance of the first oscillator if the whole system where at its local temperature $T_{1}=11$. Away from the peak the two curves are again indistinguishable.

To better understand the overall behavior of the system, in Figure 5 we represent the complementary case. There, $T_{1}=11$ and $T_{2}=1$, and the comparison is made with the case in which both oscillators were at T=11 (the monotonically decreasing curve). All of the other parameters are unchanged with respect to Figure 4. Away from k=1, once again the variance of the (now hotter) oscillator is indistinguishable from what it would be if the entire system were at T=11. The weak coupling ($k_{0}=0.01$) means that the difference in temperature has no effect on the individual oscillators, which are driven by the local temperature. Near k=1, a deep dent develops. As before, at exactly k=1 the (dimensionless) variance of the first oscillator almost equates $\frac{T_{1}+T_{2}}{2}=6$ (the minimum achievable).

**Figure 6:**
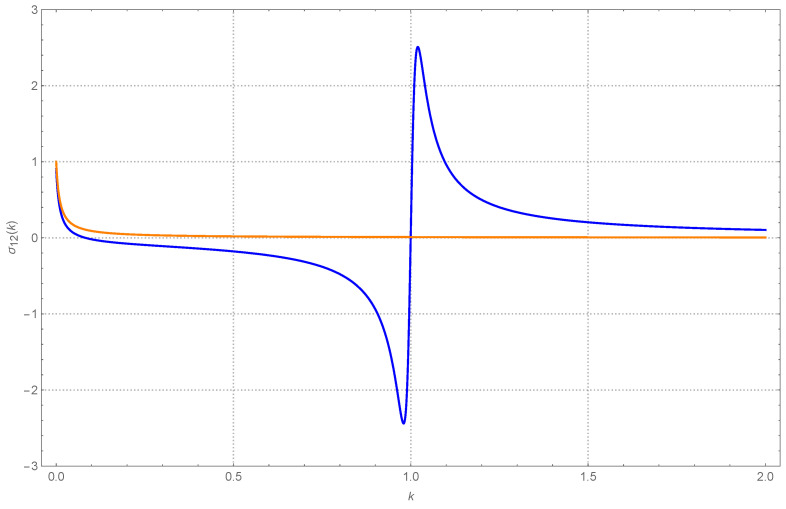
The dimensionless (stationary) covariance proper, $\sigma_{12}=\sigma_{21}=\langle u_{1}u_{2}\rangle$. The parameters are as in Figure 4. The blue curve, of sigmoid shape, is for $T_{1}=1$ and $T_{2}=11$. The orange curve, almost flat at the value 0, is for equal temperatures. The “spurious” behavior near k=0 reflects the fact that when $k_{2}\ll K$, the first oscillator is almost without any boundary interaction and only interacts with the second oscillator via their coupling *K*.

We do not report its explicit expression, but in Figure 6, we also plot the behavior of $\sigma_{12}=\sigma_{21}=\langle u_{1}u_{2}\rangle$. Across almost the whole range, when $T_{1}=T_{2}$, $\sigma_{12}≃\sigma_{21}≃0$. As one would expect, the peculiar behavior out of equilibrium, observed previously for the diagonal terms, has a counterpart in the behavior of $\sigma_{12}$. A sigmoid-like behavior appears in the vicinity of k=1. In fact, non-zero cross correlations often appear in the presence of heat flow. Very broadly speaking, comparing Figure 4 and Figure 6, we could say that $\sigma_{12}$ shows a qualitative behavior reminiscent of the negative of the first derivative of $\sigma_{11}$.

In summary, for the mentioned choices of parameters, near and away from k=1 we see two distinctive regions, affecting the overall covariance matrix elements.

**Figure 7:**
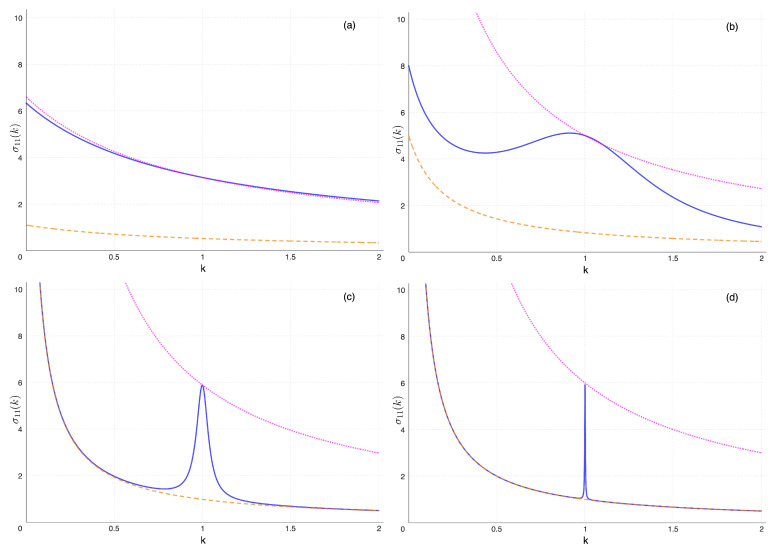
Changing the coupling $k_{0}=K/k_{2}$, decreasing from left to right and top to bottom. The dimensionless stationary $\sigma_{11}$ as a function of the parameter *k*. q=8000 in all figures. Everywhere: the blue full curve is for $T_{1}=1$ and $T_{2}=11$; for comparison, the dashed orange line (monotonic, bottom) is for $T_{1}=T_{2}=1$; still for comparison, the dotted magenta line (monotonic, top) is for $T_{1}=T_{2}=6$, the average temperature. (**a**) $k_{0}=10$: due to the strong coupling, the first oscillator behaves as if thermalized with the second oscillator, being both at the average temperature $(T_{1}+T_{2})/2$. (**b**) $k_{0}=0.25$: $\sigma_{11}$ is in an intermediate range between $T_{1}$ and the average temperature, and it is not monotonic anymore; the peak starts to develop. (**c**) $k_{0}=0.02$, the variance resonance can be appreciated. (**d**) $k_{0}=0.001$, the peak is extremely narrow: everywhere but in the closest proximity of k=1, the first oscillator behaves as if the whole system were at $T_{1}$, while very near k=1 it oscillates as if thermalized with the other oscillator.

**Figure 8:**
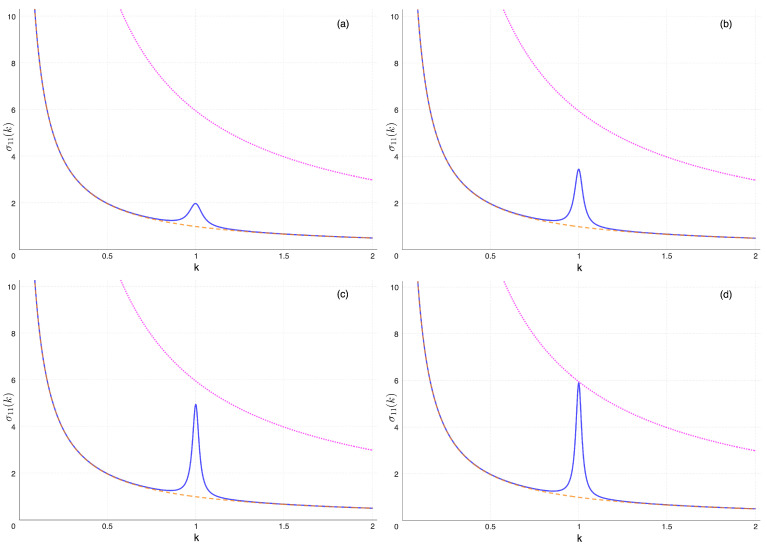
Changing the quality factor $q=Q_{2}$, increasing from left to right and top to bottom. The dimensionless stationary $\sigma_{11}$ as a function of the parameter *k*. The coupling is $k_{0}=0.01$ in all figures. Everywhere: the blue full curve is for $T_{1}=1$ and $T_{2}=11$; for comparison, the dashed orange line (monotonic, bottom) is for $T_{1}=T_{2}=1$; still for comparison, the dotted magenta line (monotonic, top) is for $T_{1}=T_{2}=6$, the average temperature. For very low *q*, the blue and orange curve coincide. (**a**) q=50, the peak starts to emerge. (**b**) q=100. (**c**) q=200. (**d**) q=1000 (saturation). Further increasing *q* produces no change with respect with the graph in (**d**).

To have a better grasp of what happens in the overall parameters space, we have considered two cases. In the first one, *q* is kept fixed at some large value, and the coupling $k_{0}=K/k_{2}$ is taken to vary from a relatively large to a very small value. This case is illustrated in Figure 7. In the second instance, $k_{0}$ is kept fixed at a small value, and *q* is gradually increased. This one is illustrated in Figure 8.

In Figure 7, we have first chosen two curves as standards of comparison. The first one, monotonic and at the bottom, represents $\sigma_{11}\left(k\right)$ when $T_{1}=T_{2}=1$, precisely the same as in Figure 4. The second one, monotonic and at the top, represents $\sigma_{11}\left(k\right)$ when $T_{1}=T_{2}=6$. The value 6 is none other than the average temperature between 1 and 11. Lagging in between, we plot the actual $\sigma_{11}\left(k\right)$ in the presence of the usual temperature imbalance. In this instance, q=8000 all throughout. Looking at the subfigure (a) on the top left, where the coupling $k_{0}=10$ is large, we see that across the whole range, the *T*-imbalanced $\sigma_{11}\left(k\right)$ is almost indistinguishable from a system in equilibrium at the average temperature. On the contrary, if we gradually decrease $k_{0}$, we see the gradual development of the peak, which becomes more localized the smaller $k_{0}$. In fact, perhaps contrary to intuition, it is $k_{0}$, and not *q*, to mostly influence the width of the peak.

In Figure 8, we have plotted curves with the very same meaning as in Figure 7. The difference is that this time, $k_{0}=0.01$ all throughout. From Figure 8a–d, we see the effect of increasing *q* from 50 to 1000. Therefore, when *q* is sufficiently small, $\sigma_{11}\left(k\right)$ is essentially the equilibrium curve at $T=T_{1}=1$. This is a reflection of the fact that the coupling is very small. Increasing *q* further and further, the peak develops and becomes ever more pronounced. It is interesting to notice that at some point it reaches saturation. Even at k=1, $\sigma_{11}$ cannot exceed the average temperature. It is of notice that increasing *q* further than in Figure 8d seems to have no effect on the width of the peak either. We also mention that a similar phenomenology affects most other elements of the covariance matrix, not reported here. More specifically, plots similar to Figure 4, Figure 5, Figure 6 and Figure 7 can be obtained for the other variables, and the other diagonal elements (i.e., the variances of the velocities), display peaks whenever the displacements’ variances do.

In conclusion, it is the combined influence of a large quality factor and small coupling which ultimately drives the phenomenon which we have named variance or covariance resonance.

In Section 6, we shall illustrate some explicit calculations with the aim of seeing some of the above results explicitly. More specifically, we shall consider some simplified expressions for $\sigma_{11}\left(k\right)$, which arise when some extremal values of the parameters are assumed.

We note that, during the revision process of this article, we have discovered that the general case of Equation (27) for an arbitrary number of oscillators has been considered in [19], leading to a formally explicit expression for σ. The variance resonance, object of the present work, has not been investigated there, but the explicit formulas given at the outset, for the case of two oscillators, are consistent with ours. Notice that arguments are offered in [19] to justify the definition of $(T_{1}+T_{2})/2$ as the effective temperature for the system (regardless of any value of the parameters). This reinforces the significance that we observe of a sharp transition, from local equilibrium (individual oscillators at their local temperatures) to global stationarity (both oscillators at the effective temperature), and back, just by finely tuning *k*.

### 5.2. Energy Flow

Following [20,21] and an adaptation in [19], we can define the energy flow $\varphi_{i}$ from each bath to the attached oscillator as

(32) $$\varphi_{1}=\frac{\gamma }{m}\left(k_{B}T_{1}-m\langle {\dot{x}}_{1}^{2}\rangle \right)=-\varphi_{2}=-\frac{\gamma }{m}\left(k_{B}T_{2}-m\langle {\dot{x}}_{2}^{2}\rangle \right)$$

Turning to our dimensionless quantities, spelled out from Equations (10)–(20), and recalling that $q=Q_{2}$,

(33) $$\begin{array}{ccc} & \varphi_{1}=\frac{k_{2}\;L^{2}}{q}\sqrt{\frac{k_{2}}{m}}\;\left(T_{1}-\sigma_{33}\right)=\frac{m\;L^{2}}{q\tau^{3}}\left(T_{1}-\sigma_{33}\right) & \\ & \varphi_{2}=\frac{k_{2}\;L^{2}}{q}\sqrt{\frac{k_{2}}{m}}\;\left(T_{2}-\sigma_{44}\right)=\frac{m\;L^{2}}{q\tau^{3}}\left(T_{2}-\sigma_{44}\right) & \end{array}$$

Notice that the fluxes are time derivatives of energy terms, and that by multiplying Equations (33) by τ, one would obtain the corresponding fluxes as derivatives in the dimensionless time domain of *s*.

An alternative approach is that to consider the mechanical energy flow between the two particles. Following [22,23,24], we might define the left-to-right flux as

(34) $$\phi =K\langle {\dot{x}}_{1}x_{2}\rangle =-K\langle x_{1}{\dot{x}}_{2}\rangle$$

which again can be recast as

(35) $$\phi =\frac{k_{0}\;k_{2}\;L^{2}}{\tau }\;\sigma_{14}=-\frac{k_{0}\;k_{2}\;L^{2}}{\tau }\;\sigma_{23}$$

Now, first notice from (28) that (consistently) $\sigma_{14}=-\sigma_{23}$. Then, the following relation is found to hold:

(36) $$\sigma_{33}-T_{1}=q\;k_{0}\;\sigma_{23}$$

Substituting (36) into Equation (35), we obtain

(37) $$\phi =\frac{k_{2}\;L^{2}}{q\;\tau }\;\left(T_{1}-\sigma_{33}\right)=\frac{k_{2}\;L^{2}}{q}\sqrt{\frac{k_{2}}{m}}\;\left(T_{1}-\sigma_{33}\right)$$

since $\tau =\sqrt{m/K_{2}}$. Therefore, the definitions (32) and (34) coincide, unsurprisingly, since the interaction is purely harmonic (see [25] for why the definition (34) is more problematic for other potentials and [21] for other anomalies regarding long chains of identical harmonic particles).

**Figure 9:**
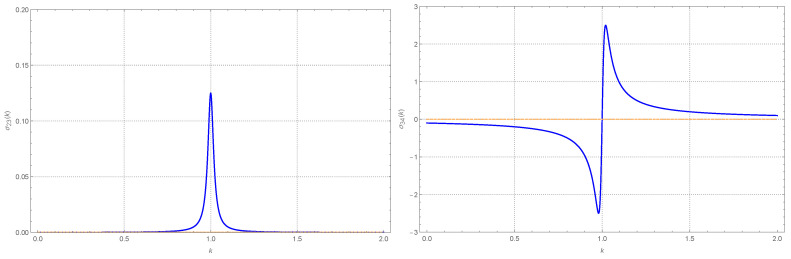
The parameters are the same as in Figure 4. Blue solid lines: $T_{1}=1$ and $T_{2}=11$. Orange dashed lines: $T_{1}=T_{2}=6$. (**Left**). The dimensionless energy flux $\sigma_{23}=-\sigma_{14}$, from 2 to 1, as a function of $k=k_{1}/k_{2}$. The flux is trivially zero when $T_{1}=T_{2}$. The flux in the opposite direction is opposite in sign, i.e., equal to $\sigma_{14}$. (**Right**). The dimensionless velocity correlator $\sigma_{34}=\langle {\dot{u}}_{1}{\dot{u}}_{2}\rangle$.

In light of the previous results, we studied $\sigma_{23}=-\sigma_{14}$ as a dimensionless measure of the absolute value of the energy flow, representing in turn the flow from the stochastic (hot) bath at $T_{2}$ to the second particle, from the second to the first particle and from the latter to the (cold) bath at $T_{1}$. In particular, we considered such quantity when we witnessed the variance resonance. The result is presented in Figure 9 Left. As it can been seen, no flow in present when $k=k_{1}/k_{2}$ is away from the value 1, consistently with both particles apparently equilibrating at their respective bath temperatures. Near k=1, we witness a peak in the flow. At k=1, the whole system, including the individual oscillators, behave as if equilibrated at the effective temperature, or mean temperature. We point out that we also found that, when the coupling $k_{0}$ is large, e.g., as in Figure 7a, the energy flux is a nonzero constant independent of *k*. Conversely, if $k_{0}$ and *q* are both sufficiently small, the flux is almost zero for any *k*.

The explicit expression is given (recalling the definition of E in (29)) by

(38) $$\sigma_{23}=\frac{2qk_{0}\left(T_{2}-T_{1}\right)}{E}$$

Finally, it can be shown that detailed balance is broken when the quantities $\sigma_{23}$ and $\sigma_{34}$, i.e., the off-diagonal terms involving at least one velocity, are non-zero. This fact is not surprising, since these quantities are the non-zero elements of the covariance matrix that involve the velocities, which change under time reversal. Formally following [26], a necessary and sufficient condition for detailed balance to hold is given by

(39) $$BB^{T}-A\sigma -\epsilon A\epsilon \sigma =0\;\mathrm{or}\;\sigma A^{T}-\epsilon A\epsilon \sigma =0$$

where ϵ is a signature diagonal matrix, with diagonal elements (1,1,−1,−1). A, B and σ are given in Equations (24) and (28). After some elementary algebra, we found

(40) $$\left(\begin{array}{cccc} 0 & 0 & 0 & 0\\ 0 & 0 & 0 & 0\\ 0 & -\sigma_{23} & -qk_{0}\sigma_{23} & \sigma_{34}\\ \sigma_{23} & 0 & \sigma_{34} & qk_{0}\sigma_{23} \end{array}\right)=0\;\;\left(\mathrm{condition}\;\mathrm{for}\;\mathrm{detailed}\;\mathrm{balance}\right)$$

and the condition approximately holds away from the peaks. Indeed, in Figure 9 Right we plot the velocity correlator $\sigma_{34}=\langle {\dot{u}}_{1}{\dot{u}}_{2}\rangle$ as a function of *k*. Taken together, the two plots in Figure 9 are an explicit indicator of the violation of detailed balance near the condition k=1. The explicit expression for $\sigma_{34}$ reads

(41) $$\sigma_{34}=\frac{q^{2}\left(k^{2}-1\right)k_{0}\left(T_{2}-T_{1}\right)}{E}=\frac{q\left(k^{2}-1\right)\sigma_{23}}{2}$$

### 5.3. An Electrical Analog

**Figure 10:**
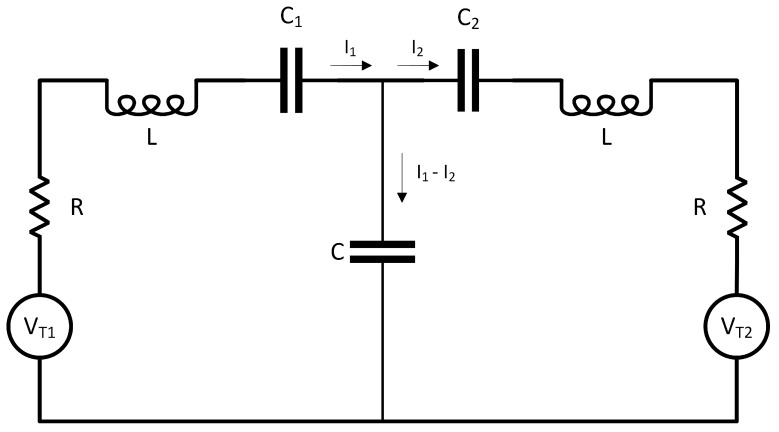
Electrical realization of our oscillators’ system. $V_{T1}$ and $V_{T2}$ are Gaussian white-noise stochastic forcing at the temperatures $T_{1}$ and $T_{2}$, given by $V_{Ti}=\sqrt{D_{i}}\;\xi_{i}$.

Recently, a loose analogy has been proposed between the behavior of a Brownian gyrator, under certain conditions, and the well known phenomenon of impedance matching [27]. The model discussed in [27], which considers two capacitively coupled RC circuits in contact with two thermal baths at different temperatures, is very similar to ours. It differs from ours in that it treats, as it is often the case in the current and past literature, the equivalent of an overdamped approximation of our model. Since we have considered the second-order underdamped case, we propose an electrical realization of our system. This can be performed by considering the circuit in Figure 10, composed of two capacitively coupled RLC circuits, each one subject to Johnson noise at two different temperatures. Outside the context of stochastically driven oscillators, the same electrical analog of mechanical oscillators has been already considered in [28].

Given $\left(q_{1},q_{2}\right)$ the circulating charges, $V_{T1}=\sqrt{D_{1}}\;\xi_{1}$ and $V_{T2}=\sqrt{D_{2}}\;\xi_{2}$ the noise voltages, the circuitry differential equations read

(42) $$\begin{array}{c} \left\{\begin{array}{c} L{\ddot{q}}_{1}=-\frac{1}{C_{1}}q_{1}-R{\dot{q}}_{1}-\frac{1}{C}\left(q_{1}-q_{2}\right)+\sqrt{D_{1}}\xi_{1}\\ L{\ddot{q}}_{2}=-\frac{1}{C_{2}}q_{2}-R{\dot{q}}_{2}-\frac{1}{C}\left(q_{2}-q_{1}\right)+\sqrt{D_{2}}\xi_{2} \end{array}\right. \end{array}$$

The analogy with Equation (8) is immediately apparent. One needs make the following identifications:

$$q_{i}⟷x_{i};\;L⟷m;\;C_{i}⟷k_{i}^{-1};\;C⟷K^{-1};\;\gamma ⟷R.$$

Therefore, given a temperature imbalance, the phenomenon of variance resonance (a sharp peak in $\langle q_{i}^{2}\rangle$ or $\langle I_{i}^{2}\rangle$ for the cold resonator) is observed when $C_{1}≃C_{2}$, provided $C\gg C_{i}$ and $L\gg C_{i}R^{2}$ (the two RLC resonators have high quality factors).

## 6. Some Limiting Cases

With the space of parameters being somewhat large, some care should be taken when considering limiting cases. We start off by simplifying the expression for $\sigma_{11}\left(k\right)$, which is given in Equation (29). Define the following quantity:

(43) $$\sigma_{11}^{\infty }\left(k\right)=\lim_{q\to \infty }\sigma_{11}\left(k\right)$$

From (29), we obtain

(44) $$\sigma_{11}^{\infty }\left(k\right)=\frac{{\left(1-k\right)}^{2}\left(1+k_{0}\right)T_{1}+\left(\left(3-k\right)T_{1}+\left(1+k\right)T_{2}\right)k_{0}^{2}+2\;k_{0}^{3}\left(T_{1}+T_{2}\right)}{\left({\left(1-k\right)}^{2}+4\;k_{0}^{2}\right)\left(k+\left(1+k\right)k_{0}\right)}$$

This expression can also be rearranged such that

(45) $$\sigma_{11}^{\infty }\left(k\right)=\sigma_{11}^{\infty ,e}\left(k\right)+\sigma_{11}^{\infty ,n}\left(k\right)$$

where

(46) $$\begin{array}{ccc} & \sigma_{11}^{\infty ,e}\left(k\right)=\frac{\left(1+k\right)T_{1}}{k+\left(1+k\right)k_{0}} & \end{array}$$

(47) $$\begin{array}{ccc} & \sigma_{11}^{\infty ,n}\left(k\right)=\frac{\left(1+k+2\;k_{0}\right)k_{0}^{2}\left(T_{2}-T_{1}\right)}{\left({\left(1-k\right)}^{2}+4\;k_{0}^{2}\right)\left(k+\left(1+k\right)k_{0}\right)} & \end{array}$$

are, respectively, an equilibrium term at $T_{1}$, and a nonequilibrium term, which depends on the difference of temperatures.

By more closely inspecting Equations (44) or (47), we can advance the following considerations. Suppose k≃1 and $k_{0}\ll 1$. Then, the only nontrivial term is the following term in the denominator:

(48) $${\left(1-k\right)}^{2}+4\;k_{0}^{2}$$

Such a term is formally comparable to the typical term in the denominator of a Lorentzian. It is minimum, and therefore $\sigma_{11}^{\infty }\left(k\right)$ maximum, at k=1. On the other hand, $k_{0}$ modulates the width near the maximum. By following through with this analogy, the value 1 is essentially the resonance value, and $k_{0}$, the weak coupling, plays the role of the damping factor. This observation partly helps to explain what we observed in Figure 7. This analogy should not be taken too literally, since neither Equation (44) nor (47) are truly Lorentzian. In fact, the maximum of $\sigma_{11}^{\infty }\left(k\right)$ is bounded. More explicitly, consider the value $\sigma_{11}^{\infty }\left(k=1\right)$,

(49) $$\sigma_{11}^{\infty }\left(1\right)=\frac{\left(1+k_{0}\right)\;\left(T_{1}+T_{2}\right)}{2\;\left(1+2\;k_{0}\right)}$$

From the latter Equation (49), for $k_{0}\ll 1$ we easily recover

(50) $$\sigma_{11}^{\infty }\left(1\right)≃\frac{T_{1}+T_{2}}{2}\;k_{0}\ll 1$$

i.e., the average temperature. This explains the value observed at k=1 all throughout our plots, when *q* and $k_{0}$ are, respectively, sufficiently large and small.

Conversely, for $k_{0}\gg 1$, i.e., strong coupling, we obtain

(51) $$\sigma_{11}^{\infty }\left(1\right)≃\frac{T_{1}+T_{2}}{4}\;k_{0}\gg 1$$

Under such conditions, we expect $\sigma_{11}^{\infty }\left(k\right)$ to be approximately the same as that of an equilibrium system with $T=\frac{T_{1}+T_{2}}{2}$. Indeed, from Equation (30), supposing $k_{0}\gg 1$, at equilibrium, we obtain

(52) $$\sigma_{11}^{eq}\left(k\right)≃\frac{T}{k+1}\;k_{0}\gg 1$$

which is consistent with (51) for k=1.

## 7. Discussion

We have considered two harmonic oscillators, each independently in contact with a stochastic thermostat. Each of the two oscillators is tied to its rest position by an elastic force, $k_{1}$ and $k_{2}$, respectively, but it also concurrently interacts elastically with the other one (the coupling *K*). We have considered the case of the two thermostats being at the same temperature, the equilibrium case, and at different temperatures, the nonequilibrium case. In the latter case, the covariance matrix may display a particularly interesting behavior. This happens when the two oscillators have both a high quality factor (the underdamped scenario) and a weak coupling. In such situations, the variance of the displacement of each oscillator from its rest position develops a peak (in the case of the colder element) or a deep hollow (for the hotter element) when the respective elastic constants are comparable in strengths, i.e., around $k_{1}/k_{2}=1$. Away from such a condition, each oscillator behaves as if the whole ambient were at its local temperature (somewhat in local equilibrium). This resonant behavior is manifest also in the non-diagonal covariance elements, which become nonzero only when the two elastic constants are comparable.

By exploring some part of the parameters’ space, we were able to distinguish the separate contributions of the coupling and of the damping. Take, for example, the oscillator at the lower temperature. By increasing the quality factor, the peak of its variance resonance increases in height, until a point of saturation. Decreasing the (already weak) coupling has instead the effect of narrowing the peak. Therefore, the weak elastic coupling *K* somewhat plays the role which is played by the small dissipation ratio, in traditional resonance problems of low-loss oscillators.

Away from the conditions of weak coupling and pronounced underdamping, the variance resonance effect disappears, and the system of the two oscillators behaves everywhere as if in thermal equilibrium at the average temperature. Despite its simplicity and smallness of size, our model seems to show both the possibilities of existence of local equilibrium, and of non existence thereof, by an opportune tuning of the parameters.

Our findings may have some importance in precision measurements, in the presence of temperature gradients and almost identical oscillators. While, in principle, $k_{1}$ and $k_{2}$ can be assumed to be known in advance, in practice, *K* may sometimes be a more obscure quantity, since it may depend nontrivially on the overall architecture of the measurement apparatus. In the presence of marked temperature gradients, depending on the parameters, one might either observe oscillations at the local temperature for each oscillator, or oscillations at an intermediate apparent temperature.

It is also worth noticing that a different (complementary) point of view can be taken. Suppose $k=k_{1}/k_{2}$ is unknown in advance, and a substantial temperature gradient is established. By tuning the other parameters, one could, say, voluntarily drive the system into the resonating territory. Then, a comparison of the average squared amplitudes of the oscillations of the two components might be used to infer the relative value of $k_{1}/k_{2}$, a posteriori.

## Data Availability

The original contributions presented in this study are included in the article. Further inquiries can be directed to the corresponding author.
